# The implications of stakeholder consultation on employee engagement: An African cross-border acquisition

**DOI:** 10.3389/fpsyg.2022.1017073

**Published:** 2022-11-03

**Authors:** Annelize van Niekerk

**Affiliations:** Department of Industrial and Organisational Psychology, University of South Africa, Pretoria, South Africa

**Keywords:** stakeholder consultation, employee engagement, authorization, diversity, psychosocial

## Abstract

**Objective:**

The objective of the study was to explore the power of stakeholder consultation on employee engagement during a cross-border acquisition in a multi-cultural context. Further, to describe the psychosocial factors at play during the employee involvement process towards enhancing employee engagement.

**Methods:**

This qualitative study presents the results from data collected in Tanzania through semi-structured interviews (46 participants) and analyzed in accordance with the hermeneutic circle and Tesch’s content analysis method.

**Results:**

The results of this study contribute to the body of knowledge to better understand the psychosocial factors at play within a multi-cultural environment which inform stakeholder consultation and will enable or hinder employee engagement. A transitional space should be created, fostering mature stakeholder engagement, promoting employee inclusion, engagement, and knowledge sharing.

**Conclusion:**

Bringing together two worlds requires building bridges to cross the cliff between contexts and overcoming diversity challenges, while incorporating diversity management in the consulting process. A multi-cultural team should be established, incorporating diversity management, applying the principles of respect, participation and transparent communication, with regular feedback on decisions made. External stakeholders in authoritative positions are not well received and should consider traditional superiority versus business hierarchy when establishing leader-follower relationships.

## Introduction

Since the dawn of globalization, the business world had to adopt an expansive view on the integration of business, technology, cultures, and people. Organizations globally have since realized how the workforce continuously changes, demanding incessant consideration towards stakeholder consultation to ensure strategic alignment of goals and objectives. Especially so when working within multi-cultural environments and with different worldviews, whilst aiming to keep employees actively engaged in their work and organizational pursuits (Islam et al., 2021). However, as Hoffmann (2018, p. 33) reminds us, we are “simultaneously citizens of the world and the local community,” which necessitates organizations to acknowledge each employee’s “individual otherness” whilst also considering their “shared human commonalities” and how that enables organizational success. It is further worth being reminded of Maslow’s (1943) hierarchy of needs, specifically level two which refers to a persons need for safety, including stability, which is often disturbed by the demands of design induced change in the workplace.

From a transactional point of view, there often seems a stronger pull within organizations to focus on the ‘hard’ challenges, such as infrastructure, and return on investment, while the ‘softer’ challenges are often given less attention or completely ignored, such as those situated in human behavior and influenced by values and cultural (including contextual) differences (Levin et al., 2012; Van Niekerk et al., 2012). Even though numerous research studies conducted over a few decades accentuate the influence of the risk involved in such inattentive and/or uninformed practices, the question remains to be answered why it is that organizations today still fail in effectively managing this risk? This disregard holds within it a significant risk to organizations and in order to mitigate this risk, it is proposed for leaders to adopt a bottom-up approach by acknowledging the power within employees and creating a platform through which they are consulted with, heard, and involved in decision making and policy design, positively influence the psychosocial factors that impact on organizational effectiveness (Haller et al., 2018; Malik and Khan, 2020). It is well known if such risk is managed, organizational commitment is enhanced, and organizations obtain a competitive advantage (Maleka et al., 2019). However, Zwikael and Smyrk (2015) propose a trust approach when an environment is high risk and turbulent, whilst adopting a control approach in a more stable environment where performance risk is perceived to be lower. Thus by adopting psychosocial factors enable the inclusion of amongst others the establishment of strong psychological contracts and trust, acknowledging all the diverse elements present in that environment that becomes the driver of change, and enables the organization to manage these factors towards ensuring employee engagement and meeting their strategic objectives (Van Niekerk, 2017; Malik and Khan, 2020; Kim and You, 2022).

**Figure 1:**
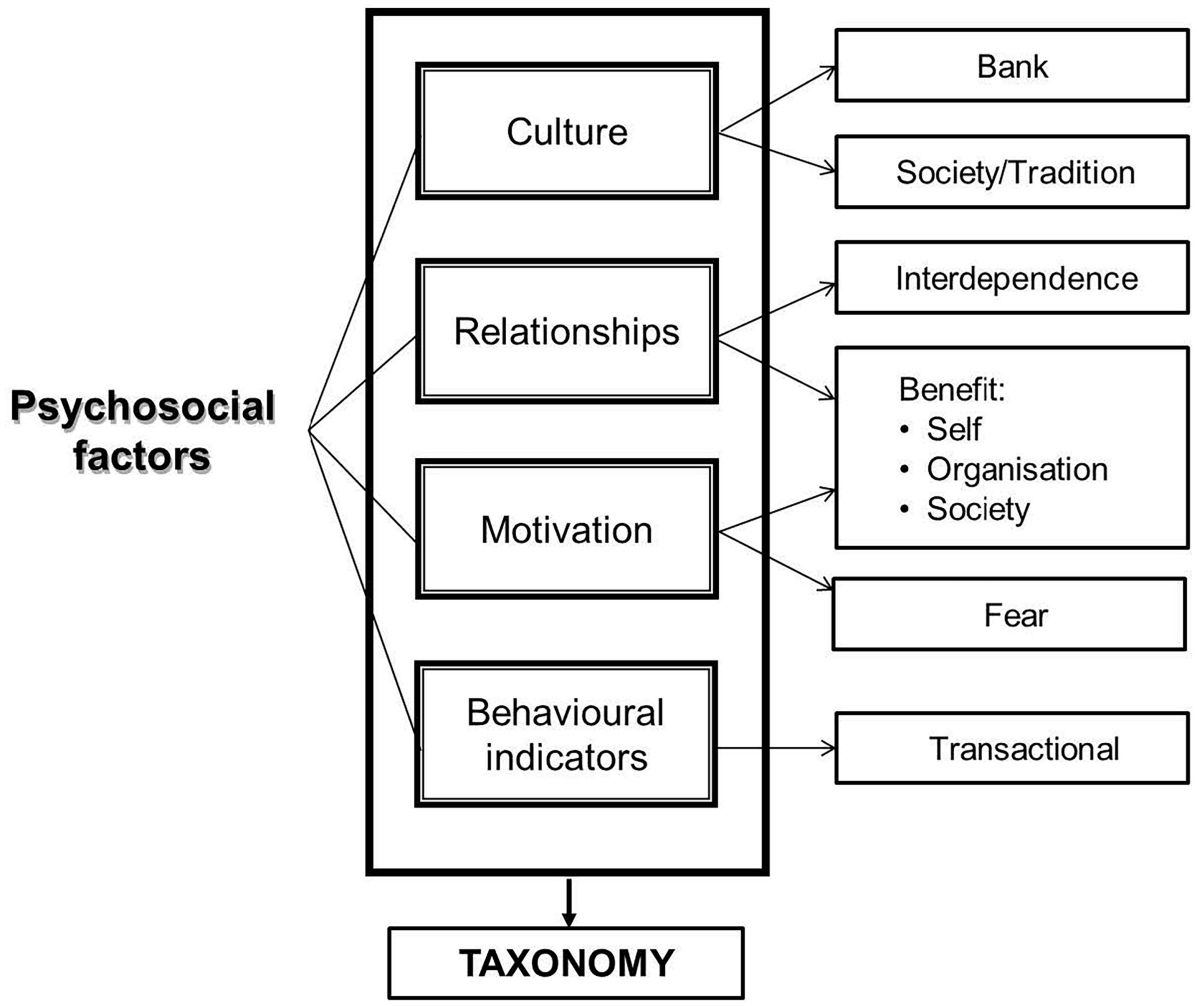
Aspects influencing psychosocial components (Van Niekerk et al., 2012).

According to Van Niekerk et al. (2012), the psychosocial components that affect change management, also in an African cross-border merger context, are primarily influenced by four aspects, namely culture, relationships, motivation and behavioral indicators (see Figure 1).

Firstly, as outlined in Figure 1 during a cross border business acquisition, the focus should be on both organizational culture and societal culture, and how these cultures regard and work with the phenomenon of stakeholder consultation. Organizational culture is clearly affected by the variations within social culture and (Cummings and Worley, 2019; Keerio et al., 2022). If this is understood, employee engagement within a multi-cultural context can be enhanced. Secondly, stakeholder engagement is also based on dependence, specifically being dependent on the relationship amongst the stakeholders with specific emphasis on interdependence and the benefit of such a relationship for the self, the organization, the immediate community, and society, thus requiring effective consultation (Cunningham, 2021). Thirdly, the ensure employee engagement, motivation which drives the different stakeholders should be well understood in relation to the self, the organization, as well as the immediate community and society. It is also important to understand this motivation can have a negative tone to it as stakeholders might experience a sense of fear as they could feel they might lose their sources of security (i.e., job security, remuneration), as well as risk their relationships with colleagues and clients (Cummings and Worley, 2019; Alolabi et al., 2021). These feelings of fear might become debilitating and result in employees disengaging and becoming less productive and not meeting strategic organizational objectives. Finally, stakeholder consultation can impact on employee engagement as behaviors might be driven by transactional features between the different stakeholders on individual, organizational and societal level in the absence or presence of clear consultation and involvement in the cross-border merger (Blom, 2018).

This research originated during a cross-border acquisition in the Tanzanian financial sector. Embracing a market-driven economy, Tanzania became a lucrative investment for international investors (Yona and Inanga, 2014; Rana et al., 2022). One such an investor was a South African financial institution, which acquired one of Tanzania’s financial institutions as a subsidiary. The acquisition required adopting new, more advanced operational risk management models as advised by the Basel Committee on Banking Supervision (Basel Committee on Banking Supervision, 2011). It is important to acknowledge, prior to this acquisition, the Tanzanian financial sector have not been familiar with the concept of risk management and specifically not with the Basel II accord on banking supervision. Also, not with organizational change management on this large scale. Following a top-down communication strategy, the management of both financial institutions adopted the Basel II advanced measurement approach as their operational risk management model and enforced it on the Tanzanian operation, ignoring all change management best practices, including consulting with employees to gain their input as to the feasibility of this new model in the Tanzanian banking context. Failing to assess organizational readiness for change to adopt this operational risk management framework resulted in local employee resistance, issues with trust arising, disengagement from the project and the new model not being implemented as successfully as was anticipated and failing in many ways.

Despite numerous studies outlining the importance of stakeholder consultation during change initiatives (Govender and Bussin, 2020), it seems organizations still have not been able to successfully master one of the key elements, that is becoming competent in how to effectively consult with stakeholders and value them as one of the key instruments towards enacting necessary change (Mann and Harter, 2016; Kajwang, 2022). As a result, many change initiatives fail because the drivers and implementers of the required change, that is the employees, are often found to distance themselves by disengaging and becoming silent spectators rather than active players towards ensuring a successful project (Govender and Bussin, 2020; Islam et al., 2021).

In this research, stakeholders are acknowledged as communities, clients, groups, and organization, but especially employees, that are affected by organizational activities or who have an influence on how such activities are influential towards achieving organizational success and impacting positively on the environment within which such an organization operates (Kujala et al., 2022; Mitchell et al., 2022). This highlights the important role stakeholder consultation plays in any organizational activity and becomes key to ensure corporate social responsibility (Fordham and Robinson, 2018). Over the years, numerous research projects have highlighted the important role effective communication and stakeholder engagement play in the success of implementing organization change including Kotter’s model on change management (Kotter, 2012; Bansal, 2015; Lozano, 2022). Stakeholders hold within them the power to oppose or support any organization activities, for example change initiatives related to systems, processes and even ownership (Cummings and Worley, 2019; Errida and Lotfi, 2020). Organization should further be well informed of the power of stakeholders, not only within the organizational structure but also within the communities and systems in the external environment such as societal culture.

Subsequently, stakeholder consultation should extend to embrace communities, clients, employees, groups, and other organizations involved in the decision-making process, should they wish to be effective. This approach is often also referred to as multi-stakeholder governance. That is the process of engaging in dialogue towards guiding, making well-informed decisions and determining an effective implementation strategy that is aligned to ensure that the interests of all stakeholders are well protected – this process gives legitimacy to the entire process or project (Gleckman, 2018; Kuenkel, 2019). When stakeholders’ environment is to be affected in any way, one cannot assume that they will accept the role of passive bystanders. Subsequently, Lozano (2022) highlights the value to be found in three specific principles worth keeping in mind towards ensuring meaningful stakeholder engagement: Firstly, that is to ensure, prior to making decisions, that the stakeholders have been given a fair opportunity to share their views on action that will impact on their environment; secondly, assurance is given that the contributions made by the stakeholders will inform decisions; and lastly, that careful consideration will be given to how the participation process is designed, that is the rules of engagement, to ensure optimal participation and impact.

Effective stakeholder consultation eliminated unnecessary delays, resistance, or possible project failures (Cummings and Worley, 2019; Lozano, 2022). Yet the struggle seems to continue towards establishing relationships characterized by complete support, cooperation and buy-in, perhaps because the decision-making power is often still seen as endemic to management and not inclusive of all stakeholders, including the employees (Heyden et al., 2017), subsequently directly impacting on employee engagement.

Employee engagement is key to organizational success. Employee engagement can be defined as an employee’s willingness to engage with and advocate for an organization and its values on an attitudinal level, including cognitively, physically, and emotionally towards meeting the strategic objectives of the organization (Lai et al., 2020; Islam et al., 2021; Jiatong et al., 2022). Numerous studies have highlighted the power of employee engagement as it activates employee support and proactiveness towards driving the organizational goals and agenda (Amor et al., 2020; Islam et al., 2021), emphasizing the need to better understand the psychological influences and drivers present within employee engagement (Ngobeni et al., 2022).

One of these influences is stakeholder consultation, as discussed earlier. The social exchange theory proposes that to effectively engage in stakeholder consultation, will result in employees exerting more effort and being more engaged to ensure the success of the project at hand. This can be ascribed to the fact that employees feel supported and valued, relationships are strengthened, and their wellbeing is taken care of (Meira and Hancer, 2021; Tauetsile, 2021).

Through their research, Blom (2018) acknowledge that certain drivers are present which enable employee engagement, but warn these drivers are context specific and function at various levels. Some of these drivers typically include the nature of work; meaningful and purposeful work; developmental opportunities; reward and recognition; healthy and productive relationships; inspiring leadership; and then most important, employee consultation, as employees want to be heard and know they have a voice (Blom, 2018; Govender and Bussin, 2020).

Organizations who succeed in establishing an inclusive organizational culture in which each employee’s participation is valued and employee engagement is ensured, will gain an advantage over their competitors and ensure organizational strategy is achieved (Goswami and Goswami, 2018). Various forms of diversity are present in an organization and can include demographic diversity such as age (generational differences), gender and race. According to the social identity theory, people also use diversity elements, such as those noted above, to categorize and group themselves into groups of affiliation (Tajfel, 1982). This theory postulates how an employee’s identity within a specific group is influenced by an organization’s actions and policies and driven by diversity characteristics and perceptions of inclusion (Hogg, 2016). Having a solid understanding of the diversities present in the environment one consults in, will enhance the effectiveness of the process of stakeholder engagement and result in stronger employee engagement leading to increased productivity and innovation (Cummings and Worley, 2019).

In the African context, certain socio-cultural parameters around gender and age are still very prominent and impact on engagement in the workplace. Knowledge of traditional socio-economic molds are vital to understand the interaction amongst men and women and between different age groups. Traditionally, in Tanzania and many other African countries, women had, and in many cases still have, a very specific place in society, which mostly do not include being educated or accepted in the business world. Yet, in business this traditional mold seems to be challenged as women are emerging by upskilling themselves in specific business domains and entering strategic, even management positions, in business, where men now often report to women (Van Niekerk et al., 2012; Van Niekerk, 2017). However, in the African cross-border merger context, research on this topic seems absent. Differences amongst generations should also be considered with further research as it is evident how a younger generation might respond differently than older generations to certain contexts. The Uhuru generation in Tanzania is a good example if one considers how they push behavioral boundaries like the Western Generation X, regarding social, political, and moral issues (Ntarangwi, 2013; Zeleza, 2014).

Diversity is also found in affiliations to different groups. One example true to the African context is even though a group of employees working for the same organization are all Tanzanians, they emanate from different rural areas and tribes, speaking different languages (up to 129 different languages are spoken in Tanzania; Ethnologue: Languages of the world, 2014) and practicing different socio-cultural and religious beliefs. Diversity is also found in the level of skill and knowledge amongst employees, as well as relationships between leaders and followers (Zoogah and Beugré, 2013). Tertiary institutions in some fields of study such as banking and risk management do not yet offer formal qualifications in these specialty areas, resulting in the younger generation going to universities in America and Europe to qualify themselves and obtain the necessary skills and knowledge. As a result, these young professionals are exposed to and become more aware of global influences, making them more receptive to the value of change and embracing the unknown when returning to their native countries, resulting in conflict with the more tenured employees who do not have such qualifications or exposures (Van Niekerk, 2017). This is especially the case where the higher qualified younger employees are appointed into leadership positions and have older, unqualified, and less skilled followers reporting to them. This results in paradigmatic conflicts and psychosocial differences which negatively impact on leader-follower relationships and subsequently result in change failures (Van Niekerk et al., 2012; Zoogah and Beugré, 2013; Zeleza, 2014; Van Niekerk, 2017).

During a cross-border acquisition, stakeholder consultation becomes a complex task and process with numerous layers and enablers, especially on a psychosocial level. Within the African multi-cultural and diverse environment, great care should be taken to consider different worldviews, as well as differences on cultural, generational, socio-economic, skill and knowledge, gender and leadership levels, to name just a few. Thus, in their quest to add new, innovative organizational development capabilities, organizations should adopt a multi-view approach towards ensuring employee engagement during the stakeholder consultation process. In the milieu of this study, paradigmatic worldview assumptions between Europe, South Africa and Tanzania should have been considered to underpin the worldview of all stakeholders in this diverse context, yet it was neglected (Chigwendere and Louw, 2018).

Engagement and consultation with all stakeholders during a cross-border business should consider for cultures are predominantly individualistic or collectivistic, for example compared to the South African culture being predominantly individualistic, Tanzanians appear to predominantly have a traditional collectivist culture (Van Niekerk et al., 2012; Dennehy, 2015). Considering Hofstede’s (1994) definition of collectivism, and the Tanzanian environment, personal interest is subordinate to that of the larger group, with a strong emphasis on sharing, cooperation, group harmony, greater concern with group welfare, and sometimes antagonism towards outsiders, such as another cross-border organization entering. Within industrial psychology the collectivism and individualism band are used as an effective measure to evaluate value differences across cultures, and important element to be used during stakeholder consultation and engagement (Schwartz, 2014; Taras et al., 2014). Due to the African collectivist culture, boundaries between the self and other, or the private and communal sphere, are porous and directly impacts on what motivates and drives stakeholders’ behavior within an organization and within the relationships between stakeholders, especially employees (Okeke et al., 1999; Olausson et al., 2009) and therefore warrants special consideration.

As noted earlier and supported by Vanhala and Dietz (2019) and Xu et al. (2016), building healthy trust relationships where employees feel their views and experience are acknowledged and valued through stakeholder consultation, will positively impact on employee’s engagement. However, establishing such interpersonal trust relationships is often challenged by intercultural communication differences (Sułkowski, 2016). These differences are especially noticeable in the high culture distance present among African and European cultural interaction; however, establishing strong interpersonal trust relationships will yield improved employee engagement and organizational success (Rothmann and Rothmann, 2010; Vanhala and Dietz, 2019).

Previous research seems to be ample in the insight it provides on the strategic and financial factors contributing to the success of mergers and acquisitions; yet, it ignored the non-financial factors, such as the socio-cultural and human behavioral issues, (Stahl et al., 2013; Viegas-Pires, 2013; Weber and Tarba, 2013), and specifically so during cross-border mergers and acquisitions (Zhu and Huang, 2007; Sinkovics et al., 2011; Sacek, 2012). However, previous research specifically focusing on the power of stakeholder consultation on employee engagement during cross-border mergers seems to be wanting, especially so in the African context. Renn (2008) and Young (2006), emphasize the importance of having good insight into the psychosocial components which impact on cross-border mergers and acquisitions, to develop a robust model which effectively addresses the ‘people’ component through employee engagement as stressed by the Basel Committee on Banking Supervision’ (2011).

Considering the above, this study aimed to answer the following research questions: What is the influence of stakeholder consultation on employee engagement during a cross-border acquisition in Africa? What are the psychosocial factors present during the process of consultation, or lack thereof, which enable engaged employees?

Considering the research questions noted above, this study aimed to explore the power of stakeholder consultation on employee engagement during a cross-border acquisition in Africa. More specifically, to identify the psychosocial factors present during the process of consultation, or lack thereof, which enable or deter engaged employees. Finally, to make recommendations to organizations considering cross-border acquisitions within the African multicultural context, on consultation best practices to promote employee engagement. This will lead to optimal stakeholder consultation, increase employee engagement and ultimately reduce the risk of failure of an integrated acquisition, whilst ensuring sustainable and effective risk management.

## Materials and methods

### Research approach and strategy

This study adopted a qualitative research approach and hermeneutic phenomenological research strategy (Creswell and Creswell, 2017). Reality is constructed as participants subjectively experience and make sense of a phenomenon, that is the influence of stakeholder engagement, or lack thereof, on employee engagement (Creswell and Poth, 2018). Therefore, given the focus of this research, qualitative research allowed for a homogeneous exploration providing a broader, open-ended inquiry towards better understanding behaviors of values, beliefs and assumptions as experienced by the participants and which influence risk management, in order to create harmony (Choy, 2014).

### Participants and setting

Considering the research problem, participants were purposefully selected for theoretical reasons as they have lived through or within the phenomenon (Creswell and Creswell, 2017) and consisted of 46 employees across various levels within the organization’s head office (HO) and branches (B). This included three whites (W), 43 Africans (A), 27 males (M) and 19 females (F). Participant descriptors were applied when quoting participants verbatim to ensure anonymity and confidentiality. For example, Participant 19AFB is the 19^th^ participant, and an African female situated within a branch.

### Data collection

In line with the hermeneutic phenomenological methodology, this exploratory study collected data through interviewing 46 participants by means of semi-structured interviews, consisting of 9 primary interview questions in line with the objectives of the study, followed by further probing questions as was deemed necessary (Creswell and Creswell, 2017; Salkind, 2018). The interviews were conducted in person in Tanzania and audio recorded after permission was obtained from each participant prior to the interview. This enabled a flexible approach towards exploring the participants’ lived experiences and gaining insight into the intersubjective experience of a community of participants (Kelly, 2006). This included engaging with the participants to share their lived experience of how they were consulted with, or not, and how this impacted on their employee engagement. Consideration was also given to the researcher as instrument and the potential for interviewer bias by regularly reflecting on the researcher’s own opinions (Salkind, 2018). In line with the hermeneutic phenomenological paradigm, inductive reasoning moves from observing a specific phenomenon towards drawing a conclusion based on the specific phenomenon (Babbie, 2008). Throughout the interviews the researcher continuously reflected with senior research colleagues at the tertiary institution and members of the Tanzanian team who assisted in setting up the project, to manage bias and possible over-exaggeration to ensure the researcher maintains an authentic interpretation of the Tanzanian context and experiences (Stephens, 2009). The researcher also made use of bracketing, assisting the researcher to forget momentarily reality as known by the researcher, yet whilst also being inextricably situated in this world (Lowes and Prowse, 2001). The researcher aimed to create a safe environment for participants to share their lived experiences, by being sensitive to their religious, gender and cultural beliefs, by listening attentively, acknowledging their reality as their own subjective truth, and continuously confirming whether the researcher correctly understood what the participants were aiming to convey (Breakwell, 2012). A professional transcriber who had significant experience in transcribing interviews for research purposes, was also utilized and transcribed in all the interviews verbatim to allow for more accurate interpretation and access to exact quotations (Alvesson, 2011). Interviews were conducted in Tanzania with the assistance of translators where needed and reflective practices were employed to ensure clear, truthful understanding of the participants’ lived experiences.

### Ethical considerations

Permission was obtained from the organization to conduct the research, whereafter the researcher obtained ethical clearance from the Research Ethics Review Committee of the University of South Africa (Ref.: 2020/CEMS/IOP/019). The participants were provided with a research information sheet outlining the background and purpose of the research and their expected role. All the participants signed an informed consent form outlining all the required research ethical guidelines, such as no payment/incentives for participation, voluntary participation, protection of confidentiality and anonymity, and how results will be reported. All electronic copies of the data were password protected and hard copies were kept secure in a locked cabinet with only the researcher having access to the data.

### Data analyses

True to the hermeneutic phenomenological paradigm, data were analyzed using the hermeneutic circle to connect the parts with the whole and then incorporated in accordance with Tesch’s (1990) eight steps of content analysis as it allowed for a greater understanding of the relationship between interpretation and context, thus connecting the parts with the whole (Creswell and Creswell, 2017). The hermeneutic circle allowed for the connection of parts to a whole within the original context, which included the research setting and the interior of the researcher, that is own experiences and language as situated in our own social reality (Stephens, 2009, p.15). The content analyses steps encompass preparing the data (i.e., transcribing and organizing field notes), obtaining an overview, allocating open codes (i.e., read and searched for switches from one topic to another, asking myself what is this about and what is said), generating categories and themes by using a spreadsheet indicating comparisons between interviews and then clustering similar categories together as themes, continuing with coding (i.e., going back to the transcriptions and coding all parts again whilst still looking for new themes), describing themes and again with the use of a spreadsheet categorized the themes in major, unique and left overs and checked for duplications whereafter they were mapped into categories, interrelating themes or categories’ content were summarised and focus was placed on similarities, uniqueness, messiness or contradiction and information that might be missing, and interpretation crystalizing themes into research outputs. In addition, the services of two independent co-coders were obtained to code the first 10 interviews, using applied framework analysis (Srivastava and Thomson, 2009) to establish the researcher’s own dependability as data analyst. This triangulation process allowed establishing multiple perspectives and confirmed credibility of coding, enhancing the rigor (Cardano, 2020). Both techniques allowed for a system of circular movements between the holistic meaning of the text and its distinct parts, where the researcher continuously moves between listening and reading, doing reflective writing and expanding the interpretation (Kelly, 2006, p. 372).

This research adopted an inductive and deductive approach. As an industrial and organisational psychologist, the researcher already has knowledge of psychosocial theory and by being mindful of and consistently using this theory improved the standing of this research (Zikmund et al., 2013; Henstrand, 2015). This study was also inductive as it constructed a theoretical model from the data which integrate the psychosocial components into an ORM model (Creswell and Creswell, 2017).

The researcher also made use of bracketing to brace the researchers own preconceived philosophies or believes on this topic, eventhough in hermeneutic phenomenology the researcher is believed to be inextricably situated in his or her own world (Creswell and Creswell, 2017).

Verbatim quotes present the authentic voice of the participants, provide a descriptive account of the phenomenon of how they were consulted with or not, and act as an enabler of employee engagement (Creswell and Creswell, 2017). As described above under Participants and Setting, pseudonyms were used to protect the anonymity of participants.

## Results

This research identified four themes relating to the power of stakeholder consultation which significantly impacts on employee engagement. These themes are (1) tug of war: opposing worldviews; (2) psychological contract; (3) consult and authorize; and (4) recognizing diversity.

### Theme 1. Tug of war: Opposing worldviews

A transitional space seems to exist between the national culture of a country, as it is socially rooted in the individual, and the organizational culture. Within the African context, communal relationships are highly valued and function in line with the principles of Ubuntu and Ujamaa (Nyerere, 1968; Kumssa and Jones, 2015). Therefore, the welfare of others is important and impacts on employee engagement.

The tug of war continues as newly introduced business requirements prohibit employees from assisting clients as they have done previously. As banking clients are often also friends or family from the community, employees fear a breakdown in relationships owing to compliance to business requirements as they can no longer help clients as they used to. In the words of Participant 38AMHO, “unlike many other places, Tanzania has a very strong culture […], where we come from, I think there’s some kind of social fabric that we are connected.” Participant 8AMHO pointed out the importance of sharing in saying: “We have to share what we have.” This results in employees being challenged when a client walks into the bank to apply for a loan and expects the bank to approve the loan (because the bank has the money). However, owing to business requirements, the client perhaps does not qualify, and the loan is not granted. According to Participant 3 AFHO, employees then fear “being segregated in their community” or as Participant 8AMHO states, “being pushed out.” The dilemma remains that of the employee who faces the client as Participant 40 AMHO explained, “It is not fair because I am the one who knows the customer, not the people at head office or [in the] credit department.”

National and organizational culture should be considered and respected when consulting in multi-cultural environments (Zoogah and Beugré, 2013). Ignoring culture results in feelings of insult and disrespect, resulting in employee disengagement, or employees doubting their contributory worth to the organization. The last resort is taking industrial action, which hinders employee engagement and results in ineffective performance.

Participant 43AMHO explained how he has experienced a lack of consideration towards cultural differences resulting in employees feeling “this is an insult to us, this is not good to us.” Participant 19AFB agreed and added they feel that “top management does not trust the staff,” resulting in “the staff do not feel like they are part of the bank.” Participant 18AMHO echoed the same assertion and argued that the staff then believes the only way they can be heard is to “go through maybe a trade union,” resulting in employees disengaging from the task at hand and engaging with industrial actions.

### Theme 2. Psychological contract

When consulting, a new relationship of inclusivity and trust must be formed. As an organization is a social system, establishing a trust relationship requires employees who feel they belong and are cared for (Potgieter, 2016; Kim and You, 2022). Employees seem to desire employers who are “more committed” and feel they were not cared for as the “personal touch” was lacking, resulting in employee disengagement (Participant 41AMHO). Participant 18AMHO emphasised how “most of the people do not feel like they belong to the bank; they feel like they have to work here because you earn the salary.” Participant 31AMHO agreed, “employees [do] not feel they are part of” the organization and “are not feeling that they are cared for.” Participant 2AFHO agreed and noted, “some people in the branches – they feel like they are not part of the organization.” Participant 19AFB believed “management does not trust the staff; so, they do not tell what’s going on. Now the staff do not feel like they are part of the bank … if I belong to this family, I think if it’s a problem, I need to be involved.”

According to Participant 18AMHO, effective communication and feedback further require attention. He believed management should “bridge the gap so that communication should flow, and management should give feedback to the staff. That way they’ll feel that they belong to the bank.” Employees seem keen to contribute to the success of the organization. However, Participant 19AFB felt that “I do not know how my performance contributes to the big picture” as “communication is top-down; it’s a one-way traffic.” Management seems to own all the information and decision-making powers, which results in a “gap between the top management and the other staff,” which “is too wide.”

### Theme 3. Consult and authorize

There is no consultation with the stakeholders who are knowledgeable and protective about the dynamics of their specific environment and/or context, and who are needed to drive the project or change initiative. Not involving local stakeholders in a consultative process and authorizing them to share their knowledge to contribute towards, for example, the drafting of new policies and procedures as well as the implementation thereof in a manner which is contextually relevant, results in a lack of buy-in, increases distrust or skepticism about the new proposed initiatives and increases resistance, which ultimately leads to project failure.

As Participant 9AFHO asked, “How is that policy and/or procedure applicable to Tanzania?” Even in instances where the proposed outcome of the consulting service appears to be positive:

… they went on without consulting them, they implemented. And that one annoyed the union … they didn’t want to change … because the union was not consulted … so everything which comes they warded off. Really, even if something good is in that they say, ‘Humph, really?’ (Participant 43AMHO)

All the participants noted the importance of understanding the context, which is in this study, Africa. As Participant 2AFHO posited, they need to “study the environment around” to gain a sense of “how we are operating here” rather than coming with “westernized practices.” Foreign consultants are perceived to enter the African context, making various assumptions. As participant 30WMHO postulated, “[consultants] come in having a certain understanding of how they work in their system, and they just assume that it’s happening here. They do not even ask the question how we do it on this side?”

Participants indicate a definite willingness to learn from consultants. Yet, numerous participants pointed to the presence of infantilization in the system and indicated how especially the older generation experienced this as they felt their knowledge and experience were being ignored. Consultants placing themselves in the role of parent, treating employees like children with a commanding tone, are strongly rejected and this results in resistance from the employees as explained by Participant 2AFHO:

Not like the way you treat the children. Some children: you must do this and don’t ask questions. We are supposed to do this now. I don’t think that is the correct way … if you do that you will find some people are trying to resist and even the change process becomes very complicated.

This further results in lacking or ineffective communication platforms as Participant 18AMHO indicated: “there’s no forum; there’s no place for them to get the ideas and contributions from our staff. We have very narrowed feedback communication to the management, very narrowed.” Employees emphasize the importance of consultants acknowledging the wealth of employee knowledge and experience as Participant 38AMHO alluded “… sometimes it’s important to attach a lot of weight to the facts on the ground.” Yet Participant 19AFB warned that should one wish for “people to ‘own’ what they are doing,” information should not be merely “communicated down.” A platform should be created in which discussions can take place between all parties involved as “communication has to be two ways.”

Numerous participants agreed with Participants 2AFHO that management and consultants “should reach out more and people should be more involved. They should feel part of the big strategy.” Participant 29AMHO felt that “there needs to be more input from bottom up …” If not applied, Participant 43AMHO believed it will result in “some friction” and recalled past instances where there were a “lack of consultation from time to time, harmony in the work was not there.”

Involving employees and recognizing their needs, expectations and knowledge, according to Participant 21AFB, will result in employees “seeing it, the big picture … They feel they are part of the business; they must safeguard the business” and they will engage to an extent in which the business becomes “their life.”

### Theme 4: Recognizing diversity

Africa is uniquely diverse culturally and generationally regarding skills, gender, and leadership and these differences should be well understood.

Within the traditional socialist culture, and specifically cultural and generational differences acknowledged, behavior favors the collective and numerous participants indicated how the older generation still strongly advocates for such a culture. However, Participant 4WMHO alluded to how the younger generation, including the Uhuru generation, seems to be “much more aware of what’s happening” and wishes to “focus and drive the business.” Participant 32AMHO voiced how the younger generation understands how capitalism “can get me an extra mile.” Participant 38AMHO believed it stems from

[T]he younger generation they’ve got parents who were bureaucrats […] they served under Nyerere […] [whom] was a socialist, but honest man […] those are the guys (referring to the parents) who were serving you, very senior people, but they retired poor. Now the children of those guys, when they have an opportunity, as they look back, they can be very dangerous. They can say, ‘look, if my father who was educated, who was very honest and he lived that life which we had to endure, if I have opportunity I have to get out of that’. Now not getting out of it by being an entrepreneur but taking an opportunity.

Participant 31AMHO supported this view:

Nowadays young boys from the university, they want to come here, work for some few years with their cars, the good life, the houses, rewards – but like old guys they’ve worked for 30 years [and] leave the bank with nothing. Everyone wants to earn fast, wants to live high class or something like that. Everyone wants to grab whatever is in front of him.

Skills differences are prominent when consulting in Africa, as the available range of skills should be well understood. In some instances, skills are lacking owing to inadequate educational programmes and systems, while in others existing skills are often undermined, not acknowledged or utilized. To illustrate this point, Participant 2AFHO explained risk management:

With us it just started. But, even in universities, even in schools that we went previously. We never learned about risk. Unlike now we have a subject called risk in universities, even in secondary schools, which is not the thing here. It is not a subject at O levels, you know. But if I go to [the] UK, if I go anywhere outside Tanzania, there are subjects where risk is a subject that one needs to understand and sit down and write an examination to that effect.

On the other hand, Participant 12AFHO shared how they felt de-authorized when consultants “came to this country […] they pre-assumed that we do not have that education level that they have.” Participant 38AMHO recommended that consultants rather begin with a skills audit and assess “each area’s staffing levels” to determine “… what are the complements, what are the skills” before they start implementing changes.

Traditionally, gender differences are prevalent within the African culture. It is important to understand that, traditionally in Africa, women had specific positions in society inspired by patriarchy, chauvinism, and misogyny, which included not being educated and most certainly not being included in the business world. According to Participant 34AMHO “our culture is sort of a […] we come from sort of a male-dominated […] what we call a masculine feel for administration whereby the males tended to dominate each and everything.” This seems to have changed over time according to Participant 34AMHO who contended that, “ladies occupy senior posts in different institutions; they are going to school etc. and perhaps maybe they are brighter and more intelligent than men.” Participant 35AFHO agreed and confirmed that women are breaking the traditional mold and seem to embrace the world of business more eagerly than men and “it’s the women who are coming up ….”

Leadership differences were observed. Some participants experienced management’s behavior and lack of consultation as hindering the process of building relationships. Participant 4WMHO experienced that management came “from this extreme to the other extreme, whereby there should be a middle ground.” Participants 9AFHO and 37AFHO were of the opinion one should “customize things to fit each country specifically” and “customization [should] reflect the local environment, so that it reflects everything which is on the ground.” Alternatively, it could result in “we feel that, okay, these guys do not trust us” as Participant 4MHO explained. Or, as Participant 18AMHO described, “When they came in, the staff treated the investor, the foreigners, like enemies. So everybody […] management treats staff as enemies and staff treat top management as enemies.” In line with the socialist culture’s values and norms, Participant 12AFHO explained, “if you come with commands, instructions, ultimatums, they keep on looking at you like this and they do not do. […] But you must bring them together, you agree, you move – you agree, you move and treat them fairly, with dignity.” Both Participants 12AFHO and 13AMHO emphasized, “once you make a mistake to one of mistreatment” people will talk as “this is communal.” As a result, if you want collaboration and success, you must win the community over, “then you are up there – you fly very easily.” However, if you are in “conflict with part of the community, you are finished.” In conclusion, Participant 12AFHO believed “everybody has a good side and a bad side. But … as long as you do not push them, we will not have any problems. Come to our country; respect us. We treat you like kings. But do not come here and push us and disrespect people.”

## Discussion

**Figure 2:**
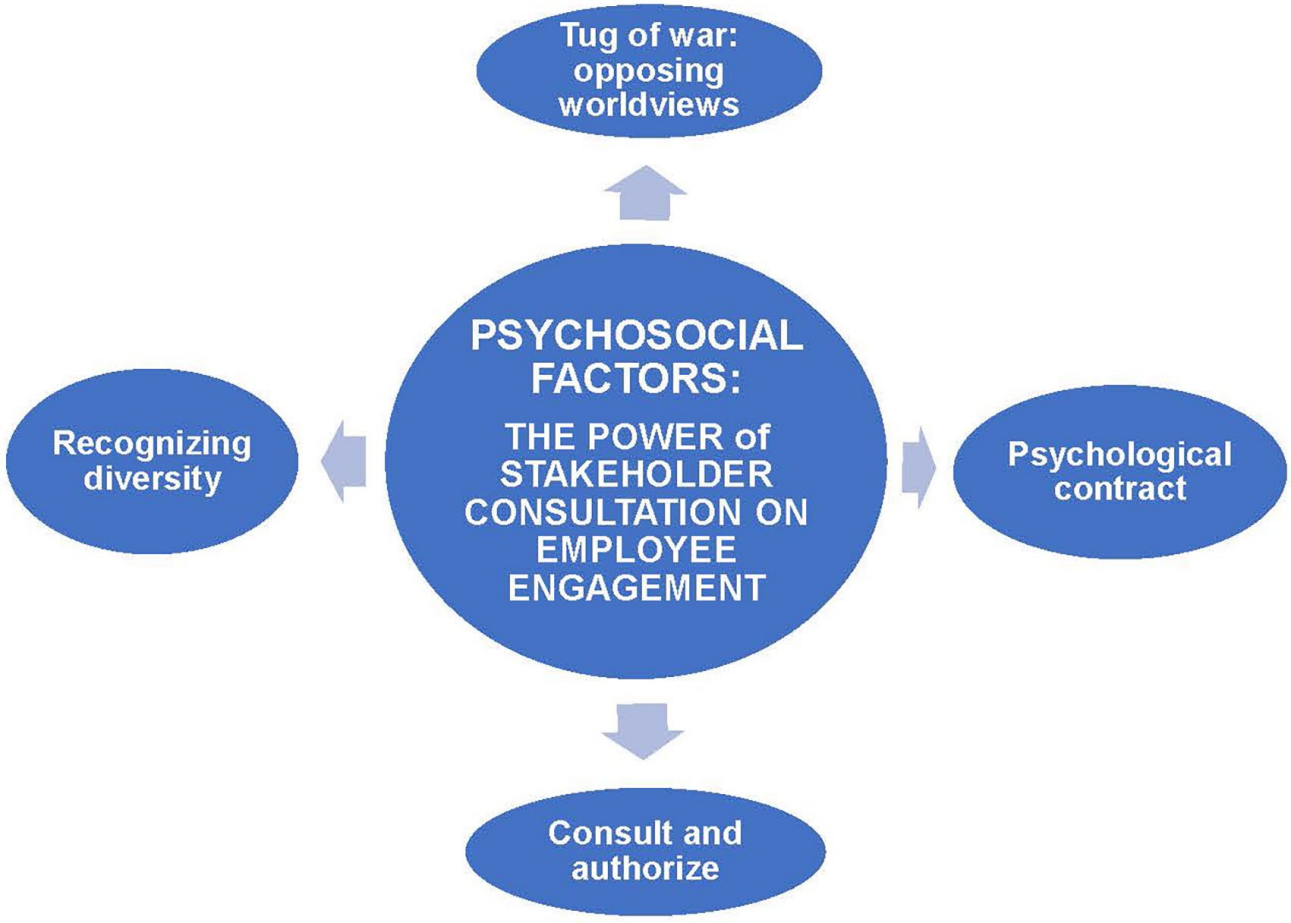
Psychosocial factors: The power of stakeholder consultation on employee engagement.

In support of the psychosocial components noted in Figure 1 and discussed earlier, which influence behavior during a change initiative, that is culture, relationships, motivation, and behavioral indicators (Van Niekerk et al., 2012), the findings of this study present four psychosocial factors, as illustrated in Figure 2, which becomes enablers and contribute theoretically and practically to the change management practice of implementing a risk management framework during a cross-border acquisition in Africa. However, psychosocial enablers have a further layer of psychosocial drivers that influence behavior and provide specific capabilities to a system (Laffort and Cargnello-Charles, 2014), but which seems to be inadequately incorporated into risk frameworks (Young, 2006). These include amongst others national culture; organisational culture; organisation as a social system; interdependence amongst stakeholders; right of existence of risk management; operational nature of risk management; management of change; and enablers of fraud (Renn, 2008; Laffort and Cargnello-Charles, 2014). Therefore, supported by literature, the findings of this research are the power behind stakeholder consultation and its ability to enhance employee engagement during a cross-border acquisition in a multi-cultural context.

In a multi-cultural context, such as Africa, a *tug of war* can become a reality because of opposing worldviews (Nyerere, 1968; Kumssa and Jones, 2015), which necessitated the importance of understanding that a transitional space should be created in which a mature stakeholder engagement process is facilitated to promote employee inclusion, engagement, and the sharing of knowledge. Employees feel this leads to weakened communal relationships as client welfare is not promoted and the organization’s image suffers in the community, which they are part of and aim to serve (Holtzhausen and Fourie, 2011; Kumssa and Jones, 2015).

By establishing a healthy *psychological contract* founded on a relationship of trust, disabling factors such as bias and labelling; ethnocentrism; unique environmental contexts; language differences; uncertainty; and different cultural values can be eliminated from the context (Chigwendere and Louw, 2018). Entering a psychological contract is important and includes establishing a relationship in which employees feel the employer trusts them and they are included in the decision-making process, rather than just being “told” what should be done, and then not being able to see the vision or bigger picture towards which they are working (Kim and You, 2022; Supriharyanti and Sukoco, 2022). Being cognizant of the presence of intercultural communication whilst consulting in a multi-cultural context can help eliminating potential communication barriers such as, but not limited to, bias and labelling; ethnocentrism; unique environmental contexts; language differences; uncertainty; and different cultural values (Chigwendere and Louw, 2018). Having a healthy psychological contract further enhances inclusivity if it can be supported with a transparent communication strategy, in which employees feel they have a voice (Holland and Scullion, 2019). Employees want to commit psychologically and feel they are given a platform to contribute towards making decisions and affecting the necessary change (Doeze Jager et al., 2021; Ngobeni et al., 2022). This will also assist in bridging the gap that seems to be experienced between management and employees (Supriharyanti and Sukoco, 2022). This will result in psychological employee commitment and engagement, bridging the gap between stakeholders as it promotes consultation (Supriharyanti and Sukoco, 2022).

To *consult and authorize*, a triad relationship should be established between conforming to policies, procedures, and frameworks; and collaboration between all stakeholders, while remaining relevant. To create harmony, all stakeholders should be consulted and the employees, from the bottom-up, should be authorized to share their wealth of knowledge and experience regarding the context (Haller et al., 2018). Instead, imbalance and disruption are caused by a flawed top-down communication strategy, which does not create platforms or opportunities to establish consultative forums. Subsequently, employees who feel the organization have become “their life” and who acknowledge that they do have things to learn from the consultant(s), are left feeling they are not given the authority to “own” the process and to engage with the contextual dynamics and share the knowledge they hold – they therefore disengage from the organization (Goswami and Goswami, 2018; Islam et al., 2021). Stakeholders indicate their wish for a mature consultation process and communication system in which tenured employees, with their wealth of experience and knowledge, are respected and part of an equal relationship, to ensure that the strategic objectives of the project are met (Errida and Lotfi, 2020; Meira and Hancer, 2021). Balance should be found between bureaucratic controls and procedures and allowing inclusive engagement by all stakeholders as this will result in full ownership taken and healthy employee engagement (Senge et al., 2014; Bansal, 2015).

Furthermore, recognizing *diversity and embracing* the value of consulting with cultural sensitivity should be well understood and collaborative solutions should be the focal point whilst all stakeholders should be open to mutual lessons that can be learned. This stakeholder relationship should blend informal and formal organizational knowledge as is owned by its employees with consultant (practitioner) knowledge. African dynamics can easily be misinterpreted. Rather than assuming all people are the same, it is important in consulting to not only acknowledge differences but also similarities.

## Limitations

This study considered the experiences of employees and did not explore the experiences of consultants who are positioned differently in this stakeholder relationships. This study therefore only presents a one-sided view, and it is recommended that further research be conducted to also hear the voices and experiences of the consultants. Gaining insight into the experiences of all stakeholders will enable a more robust understanding of all the psychosocial factors present during stakeholder consultation towards ensuring full employee engagement.

Finally, as this study was conducted in just one African country, that is Tanzania, the results do not represent the experiences of other African countries, which all have their own unique, diverse multi-cultural contexts. In light hereof, it is recommended that further studies be conducted across the entire African continent, and even globally.

### Recommendations

It is recommended to management and change agents to not merely just adopt predetermined categorical frameworks, but rather engage in a process of content analyzing indigenous employees’ responses with the aim of establishing a new psychological contract and incorporating this into the adopted risk management framework to better mitigate potential risks associated with diversity performance. Also, stakeholders should establish a triad relationship between conforming to policies, procedures, and frameworks; and collaboration between all stakeholders, while remaining relevant. Balance should be retained between bureaucratic controls and procedures and allowing inclusive engagement by all stakeholders to enhance ownership and healthy employee engagement. Finally, management and change agent should recognize and acknowledge diversity. Consulting with cultural sensitivity should be well understood to foster an environment conducive to collaboration. Finally, organizational readiness must be predetermined and stakeholder relationship should encourage blending informal and formal organizational knowledge, as is owned by its employees, with consultant (practitioner) knowledge.

Future research is also necessary to gain insight into the experiences of change agents and consultants as they are positioned differently within the stakeholder relationship and task to be executed, and have their own unique experiences and perceptions.

## Conclusion

In conclusion, when engaging in a change management initiative, incorporating the 8-step model of Kotter can help guide the process (Kotter, 2012). These eight steps include: creating a sense of urgency, forming powerful guiding coalitions, developing a vision and a strategy, communicating the vision, removing obstacles and empowering employees for action, creating short-term wins, consolidating gains and strengthening change by anchoring change in the culture.

Also, incorporating the principles of the social exchange theory will enhance efficiency (Meira and Hancer, 2021; Tauetsile, 2021). This should include establishing a multi-cultural consulting team familiar with the principles of the context, such as Ubuntu and Ujamaa in Africa. More importantly, communication barriers and socio-cultural backgrounds should be acknowledged. The principles of respect, participation and healthy open relationships should be applied. Moreover, employees need to be consulted and heard. Management must always be transparent and provide regular feedback on decisions made or actions to be taken.

Having an outsider coming in and enacting change disrupts the lives of employees. Therefore, it is essential to acknowledge all stakeholders’ increased stress levels; cultural, generational, gender and skills differences; and leader-follower relationships (traditional superiority versus business hierarchy). The importance of acknowledging and working with the psychosocial factors present in a multi-cultural context cannot be ignored. The bringing together of two worlds requires building bridges to cross the cliff between contexts and overcoming diversity challenges. Embracing diversity and incorporating diversity management as part of the consulting process and/or relationship will assist in ensuring a fruitful and sustainable relationship. Incorporating the elements of the social identity theory, employees will be better able to position themselves within this process of change amongst groups, but also within roles to form a new identity with which they can navigate through all the diversity elements and perceptions (Tajfel, 1982).

Finally, considering the research aims of this study, it can be concluded that the power of stakeholder consultation on employee engagement during a cross-border acquisition in Africa is illustrated through the four themes which emerged through this research. This research also succeeded in identifying the psychosocial factors present during the process of consultation, or lack thereof, which enable or deter engaged employees. Meeting the aims of this research enabled making recommendations to organizations considering cross-border acquisitions within the African multicultural context, on consultation best practices to promote employee engagement. This will lead to optimal stakeholder consultation, increase employee engagement and ultimately reduce the risk of failure of an integrated acquisition, whilst ensuring sustainable and effective risk management.

## Data availability statement

The raw data supporting the conclusions of this article will be made available by the authors, without undue reservation.

## Ethics statement

The studies involving human participants were reviewed and approved by Research Ethics Review Committee of the University of South Africa. The patients/participants provided their written informed consent to participate in this study.

## Author contributions

The author confirms being the sole contributor of this work and has approved it for publication.

## Conflict of interest

The author declares that the research was conducted in the absence of any commercial or financial relationships that could be construed as a potential conflict of interest.

## Publisher’s note

All claims expressed in this article are solely those of the authors and do not necessarily represent those of their affiliated organizations, or those of the publisher, the editors and the reviewers. Any product that may be evaluated in this article, or claim that may be made by its manufacturer, is not guaranteed or endorsed by the publisher.

## References

[ref2] Alolabi Y. A. Ayupp K. Dwaikat M. A. (2021). Issues and implications of readiness to change. Admin. Sci. 11:140. doi: 10.3390/admsci11040140

[ref3] Alvesson M. (2011). Interpreting interviews. Thousand Oaks, CA: Sage.

[ref4] Amor A. M. Vázquez J. P. A. Faíña J. A. (2020). Transformational leadership and work engagement: exploring the mediating role of structural empowerment. Eur. Manag. J. 38, 169–178. doi: 10.1016/j.emj.2019.06.007

[ref5] Babbie E. (2008). The basics of social research (4th ed.). London: Thomson Learning.

[ref6] Bansal A. (2015). Understanding the integration mechanisms practiced during organizational change: evidence from five M & a transactions. J. Organ. Chang. Manag. 28, 929–947. doi: 10.1108/JOCM-12-2014-0222

[ref7] Basel Committee on Banking Supervision (2011). Operational risk: Supervisory guidelines for advanced measurement approach. Available at: http://www.bis.org/publ/bcbs/196 (Accessed February 25, 2022).

[ref8] Blom T. (2018). Organizational wellness: human reaction to change. South Afr. J. Bus. Manag. 49:a2. doi: 10.4102/sajbm.v49i1.2

[ref9] Breakwell G. M. (2012). “Interviewing” in Research methods in psychology. eds. Breakwell G. M. Smith J. A. Wright D. B. (London: Sage), 367–390.

[ref10] Cardano M. (2020). Defending qualitative research: Design, analysis, and textualization, New York: Routledge.

[ref11] Chigwendere F. B. Louw L. (2018). Towards intercultural communication effectiveness (congruence) in Sino-African interactions: a theoretical perspective. Communicare: J. Commun. Sci. South. Afr. 37, 48–65. doi: 10.10520/EJC-127f203473

[ref12] Choy L. T. (2014). The strengths and weaknesses of research methodology: comparisons and complimentary between qualitative and quantitative approaches. J. Hum. Soc. Sci. 19, 99–104. doi: 10.9790/0837-194399104

[ref13] Creswell J. W. Creswell J. D. (2017). Research design: Qualitative, quantitative and mixed method approaches. 5th Edn, Los Angeles: Sage.

[ref14] Creswell J. W. Poth C. N. (2018). Qualitative inquiry and research design: Choosing among five approaches. 4th Edn, Singapore: Sage.

[ref15] Cummings T. G. Worley C. G. (2019). Organization development and change. 11th Edn, Ohio: South-Western, Cengage Learning.

[ref16] Cunningham N. (2021). Changing organizational culture through group coaching: Fact or fiction, Sage Publications: SAGE Business Cases Originals.

[ref17] Dennehy E. (2015). Hofstede and learning in higher education: an empirical study. Int. J. Manag. Edu. 9, 323–339. doi: 10.1504/IJMIE.2015.070125

[ref18] Doeze Jager S. B. Born M. P. H. van der Molen H. T. (2021). The relationship between organizational trust, resistance to change and adaptive and proactive employees’ agility in an unplanned and planned change context. Appl. Psychol. Int. Rev. 1–25. doi: 10.1111/apps.12327

[ref19] Errida A. Lotfi B. (2020). Measuring change readiness for implementing a project management methodology: an action research study. Acad. Strateg. Manag. J. 19, 1–17.

[ref20] Ethnologue: Languages of the world (2014). Tanzania. Available at: http://www.ethnologue.com/country/TZ (Accessed January 27, 2022).

[ref21] Fordham A. E. Robinson G. M. (2018). Mapping meanings of corporate social responsibility: an Australian case study. Int. J. Corp. Soc. Responsib. 3, 1–20. doi: 10.1186/s40991-018-0036-1

[ref22] Gleckman H. (2018). Multistakeholder governance and democracy: A global challenge, London: Routledge.

[ref23] Goswami S. Goswami B. K. (2018). Exploring the relationship between workforce diversity, inclusion and employee engagement. Drishtikon: Manag. J. 9, 65–89.

[ref24] Govender M. Bussin M. H. R. (2020). Performance management and employee engagement: a south African perspective. South Afr. J. Hum. Re. Manag. 18, 1–19. doi: 10.4102/sajhrm.v18i0.121

[ref25] Haller T. Belsky J. M. Rist S. (2018). The constitutionality approach: conditions, opportunities, and challenges for bottom-up institution building. Hum. Ecol. 46, 1–2. doi: 10.1007/s10745-018-9966-1

[ref26] Henstrand J. L. (2015). “Seeking an understanding of school culture: using theory as a framework for observation and analysis,” in Theoretical frameworks in qualitative research. eds. Anfare V. A. Jr. Mertz N. T. (Thousand Oaks, CA: Sage), 2–23.

[ref27] Heyden M. L. M. Fourné S. P. L. Koene B. A. S. Ansari S. Werkman R. (2017). Rethinking ‘top-down’ and ‘bottom-up’ roles of top and middle managers in organizational change: implications for employee support. J. Manag. Stud. 54, 961–985. doi: 10.1111/joms.12258

[ref28] Hoffmann W. A. (2018). “Disruptive journeys: the role of ethics in professional development,” in Paper presented at the meeting of the 4^th^ annual ethics conference of the Department of Industrial and Organisational Psychology (Pretoria: South Africa)

[ref29] Hofstede G. (1994). The business of international business is culture. Int. Bus. Rev. 3, 1–14. doi: 10.1016/0969-5931(94)90011-6

[ref30] Hogg M. A. (2016). “Social identity theory,” in Understanding peace and conflict through social identity theory. eds. McKeown S. Haji R. (Switzerland: Springer), 3–17.

[ref31] Holland D. Scullion H. (2019). Towards a talent retention model: mapping the building blocks of the psychological contract to the three stages of the acquisition process. Int. J. Hum. Resour. Manag. 32, 2683–2728. doi: 10.1080/09585192.2019.1569546

[ref32] Holtzhausen L. Fourie L. (2011). Employees’ perceptions of institutional values and employer-employee relationships at the North-West University. J. Public Aff. 11, 243–254. doi: 10.1002/pa.417

[ref33] Islam M. N. Furuoka F. Idris A. (2021). Employee engagement in organizational change initiatives: does transformational leadership, valence, and trust make a difference? Glob. Bus. Organ. Excel. Rev. Res. Best Pract. 40, 50–62. doi: 10.1002/joe.22078

[ref34] Jiatong W. Wang Z. Alam M. Murad M. Gul F. Gill S. A. (2022). The impact of transformational leadership on affective organizational commitment and job performance: the mediating role of employee engagement. Front. Psychol. 13, 1–12. doi: 10.3389/fpsyg.2022.831060, PMID: 35465551 PMC9019157

[ref35] Kajwang B. (2022). Implications of youth workforce on employee engagement in the insurance sector. J. Hum. Res. Lead. 7, 1–7. doi: 10.47604/jhrl.1437

[ref36] Keerio K. Ahmad A. R. Abbas Z. (2022). Integration model of organizational culture and succession planning in the context of higher education institutions: a brief review. Int. J. Ser. Operat. Manag. 42, 281–293. doi: 10.1504/IJSOM.2022.123352

[ref37] Kelly K. (2006). “From encounter to text: collecting data in qualitative research,” in Research in practice: Applied methods for the social sciences. eds. Blance M. T. Durrheim K. Painter D. (Cape Town: UCT Press), 285–319.

[ref38] Kim T. Y. You Y. Y. (2022). “The influence of consultant competency and consulting service quality on small-medium enterprise’s management performance,” in Cognitive computing for risk management: EAI/springer innovations in communication and computing. eds. Samanta S. R. Mallick P. K. Pattnaik P. K. Mohanty J. R. Polkowski Z. (Switzerland: Springer), 137–148.

[ref39] Kotter (2012). The 8-step process for leading change. Kotter. Available at: https://www.kotterinc.com/8-step-process-for-leading-change/ (Accessed February 11, 2022).

[ref40] Kuenkel P. (2019). Sustainability transformations: An emerging theory and practice of SDG, Potsdam, Germany: Springer.

[ref41] Kujala J. Sachs S. Leinonen H. Heikkinen A. Laude D. (2022). Stakeholder engagement: Past, present and future. Bus. Soc. 61, 1136–1196. doi: 10.1177/00076503211066595

[ref42] Kumssa A. Jones J. F. (2015). Post-independence African policy: African socialism and the organization of African unity. Public Admin. Res. 4, 12–23. doi: 10.5539/par.v4n1p12

[ref43] Laffort E. Cargnello-Charles E. (2014). Reducing the risk of fraud in financial markets: psychosocial drivers and enactment-based perspective. World J. Soc. Sci. 4, 1–13.

[ref44] Lai F. Y. Tang H. C. Lu S. C. Lee Y. C. Lin C. C. (2020). Transformational leadership and job performance: the mediating role of work engagement. SAGE Open 10:215824401989908. doi: 10.1177/2158244019899085

[ref45] Levin M. M. Van Niekerk A. Geldenhuys D. J. (2012). A call for trust in cross border business. Afr. J. Bus. Manag. 6, 9236–9242. doi: 10.5897/AJBM11.2557

[ref46] Lowes L. Prowse M. A. (2001). Standing outside the interview process? The illusion of objectivity in phenomenological data generation. Int. J. Nurs. Stud. 38, 471–480. doi: 10.1016/S0020-7489(00)00080-8, PMID: 11470105

[ref47] Lozano R. (2022). Organizational change management for sustainability, Sweden: Springer.

[ref48] Maleka M. Mpofu M. Hlatywayo C. K. Meyer I. Carr S. Parker J. (2019). Employee engagement, organisational commitment, and job satisfaction in Namibia, South Africa, and Zimbabwe: an exploratory study. J. Psychol. Afr. 29, 393–400. doi: 10.1080/14330237.2019.1647964

[ref49] Malik M. F. Khan M. A. (2020). “Tracking engagement through leader,” authentic leadership’s consequences on followers’ attitudes: a sequential mediated mode. Int. J. Public Adm. 43, 831–838. doi: 10.1080/01900692.2019.1659817

[ref50] Mann A. Harter J. (2016). The worldwide employee engagement crisis. Gallup Bus. J. 7, 1–5.

[ref51] Maslow A. H. (1943). A theory of human motivation. Psychol. Rev. 50, 370–396. doi: 10.1037/h0054346

[ref52] Meira J. V. D. S. Hancer M. (2021). Using the social exchange theory to explore the employee-organization relationship in the hospitality industry. Int. J. Contemp. Hosp. Manag. 33, 670–692. doi: 10.1108/IJCHM-06-2020-0538

[ref54] Mitchell J. R. Mitchell R. K. Hunt R. A. Townsend D. M. Lee J. H. (2022). Stakeholder engagement, knowledge problems and ethical challenges. J. Bus. Ethics 175, 75–94. doi: 10.1007/s10551-020-04550-0

[ref55] Ngobeni D. A. Saurombe M. D. Joseph R. M. (2022). The influence of the psychological contract on employee engagement in a south African bank. Front. Psychol. 13:95812. doi: 10.3389/fpsyg.2022.95812, PMID: 35983217 PMC9379290

[ref56] Ntarangwi M. (2013). “Generation X meets the Uhuru generation in East Africa,” in Generation X goes global: Mapping a youth culture in motion. ed. Henseler C. (London: Routledge), 73–90.

[ref57] Nyerere J. K. (1968). Ujamaa: Essays on socialism, Madison, WI: Oxford University Press.

[ref58] Okeke B. I. Draguns J. G. Sheku B. Allen W. (1999). “Culture, self and personality in Africa,” in Personality and person perception across cultures. eds. Lee Y. McCaauley C. R. Draguns J. G. (London: Lawrence Erlabaum), 139–162.

[ref59] Olausson E. Strafström C. Svedin S. (2009). Cultural dimensions in organizations: A study in Tanzania (unpublished master’s thesis). Luleå University of Technology, Sweden.

[ref60] Potgieter T. (2016). “Organisational culture,” in Organisational behaviour: A contemporary south African perspective. ed. Werner A. (Pretoria: Van Schaik), 36–71.

[ref61] Rana Y. S. Druckman D. Canduela J. (2022). A turning points analysis of cross-border merger and acquisition negotiations. Negot. Confl. Manag. Res. 15. doi: 10.34891/20220406-397

[ref62] Renn O. (2008). Risk governance: Coping with uncertainty in a complex world. London: Earthscan.

[ref63] Rothmann S. Rothmann S. (2010). Factors associated with employee engagement in South Africa. South Afr. J. Indus. Psychol. 36, 1–12. doi: 10.4102/sajip.v36i2.925

[ref64] Sacek A. (2012). The merger syndrome: the emotional aspect of mergers and acquisitions. New Chall. Eco. Bus. Develop., 566–575.

[ref65] Salkind N. J. (2018). Exploring research. 9th Edn., Essex, England: Pearson Education Limited.

[ref66] Schwartz S. H. (2014). “National culture as value orientations: consequences of value differences and cultural distance,” in Handbook of the economics of arts and culture. eds. Ginsburgh V. A. Throsby D. (Oxford: North-Holland), 547–584.

[ref67] Senge P. Kleiner A. Roberts C. Ross R. Roth G. Smith B. (2014). The dance of change: The challenges of sustaining momentum in learning organizations, New York, NY: Doubleday.

[ref68] Sinkovics R. R. Zagelmeyer S. Kusstatscher V. (2011). Between merger and syndrome: the intermediary role of emotions in four cross border M&A’s. Int. Bus. Rev. 20, 27–47. doi: 10.1016/j.ibusrev.2010.05.002

[ref69] Srivastava A. Thomson S. B. (2009). Framework analysis: a qualitative methodology for applied policy research. J. Adm. Gov. 4, 72–79.

[ref70] Stahl G. K. Angwin D. N. Very P. Gomes E. Weber Y. Tarba S. Y.. (2013). Sociocultural integration in mergers and acquisitions: unresolved paradoxes and directions for future research. Thunderbird Int. Bus. Rev. 55, 333–356. doi: 10.1002/tie.21549

[ref71] Stephens D. (2009). Qualitative research in international settings: A practical guide. London: Routledge.

[ref72] Sułkowski Ł. (2016). “Social capital, trust and intercultural interactions,” in Intercultural interactions in the multicultural workplace: Traditional and positive Organisational scholarship. eds. Rozkwitalska M. Sułkowski Ł. Magala S. (Switzerland: Springer), 155–171.

[ref73] Supriharyanti E. Sukoco B. M. (2022). Organizational change capability: a systematic review and future research directions. Manag. Res. Rev., 1–36. doi: 10.1108/MRR-01-2021-0039

[ref74] Tajfel H. (1982). Social psychology of intergroup relations. Annu. Rev. Psychol. 33, 1–39. doi: 10.1146/annurev.ps.33.020182.000245

[ref75] Taras V. Sarala R. Muchinsky P. Kemmelmeier M. Singelis T. M. Avsec A.. (2014). Opposite ends of the same stick? Multi-method test of the dimensionality of individualism and collectivism. J. Cross-Cult. Psychol. 45, 213–245. doi: 10.1177/0022022113509132

[ref76] Tauetsile J. (2021). Employee engagement in non-Western contexts: the link between social resources Ubuntu and employee engagement. Int. J. Cross-cult. Manag. 21, 245–259. doi: 10.1177/14705958211007874

[ref77] Tesch R. (1990). Qualitative research analysis types and software tools, London: The Falmer Press.

[ref78] Van Niekerk A. (2017). The psychosocial component of an operational risk management model: Risky business in Tanzania (unpublished doctoral dissertation). University of South Africa, Pretoria.

[ref79] Van Niekerk A. Geldenhuys D. J. Levin M. M. May M. Moalusi K. P. (2012). Implementing an operational risk management framework: psycho-social factors in Tanzania. J. Psychol. Afr. 22, 77–86. doi: 10.1080/14330237.2012.10874524

[ref80] Vanhala M. Dietz G. (2019). How trust in one’s employer moderates the relationship between HRM and engagement related performance. Int. Stud. Manag. Organ. 49, 23–42. doi: 10.1080/00208825.2019.1565092

[ref81] Viegas-Pires M. (2013). Multiple levels of culture and post M&a integration: a suggested theoretical framework. Thunderbird Int. Bus. Rev. 55, 357–370. doi: 10.1002/tie21550

[ref82] Weber Y. Tarba S. Y. (2013). Sociocultural integration in mergers and acquisitions – new perspectives. Thunderbird Int. Bus. Rev. 55, 327–331. doi: 10.1002/tie.21548

[ref83] Xu A. J. Loi R. Ngo H. (2016). Ethical leadership behavior and employee justice perceptions: the mediating role of trust in organization. J. Bus. Ethics 134, 493–504. doi: 10.1007/s10551-014-2457-4

[ref84] Yona L. Inanga E. (2014). Financial sector reforms in bank regulations and supervision and its impact on service quality of commercial banks in Tanzania. Euro. J. Bus. Manag. 6, 45–57.

[ref85] Young J. (2006). Operational risk management: The practical application of a qualitative approach. Pretoria: Van Schaik.

[ref86] Zeleza P. T. (2014). “The protracted transition to the Second Republic in Kenya,” in Kenya: The struggle for a new constitutional order. eds. Murunga G. R. Okello D. Sjögren A. (London: Zed Books), 17–43.

[ref87] Zhu Z. Huang H. (2007). The cultural integration in the process of cross-border mergers and acquisitions. International Management Review, 3, 40–44.

[ref88] Zikmund W. G. Babin B. J. Carr J. C. Griffin M. (2013). Business research methods. 9th Edn. Ohio: South-Western, Cengage Learning.

[ref89] Zoogah D. B. Beugré C. D. (2013). Managing organizational behaviour in the African context Routledge.

[ref90] Zwikael O. Smyrk J. (2015). Project governance: balancing control and trust in dealing with risk. Int. J. Proj. Manag. 33, 852–862. doi: 10.1016/j.ijproman.2014.10.012

